# Microbial isolates with Anti-*Pseudogymnoascus destructans* activities from Western Canadian bat wings

**DOI:** 10.1038/s41598-022-14223-9

**Published:** 2022-06-14

**Authors:** Adrian Forsythe, Nick Fontaine, Julianna Bissonnette, Brandon Hayashi, Chadabhorn Insuk, Soumya Ghosh, Gabrielle Kam, Aaron Wong, Cori Lausen, Jianping Xu, Naowarat Cheeptham

**Affiliations:** 1 Department of Biology, Faculty of Science, McMaster University, Hamilton, ON L8S 4K1 Canada; 2 Department of Biological Sciences, Faculty of Science, Thompson Rivers University, Kamloops, BC V2C 08C Canada; 3 Wildlife Conservation Society Canada, P.O. Box 606, Kaslo, BC V0G 1M0 Canada; 4 Present Address: Department of Genetics, Natural and Agricultural Sciences, University of the Free State, Bloemfontein, South Africa

**Keywords:** Environmental microbiology, Applied microbiology, Microbial ecology, Microbiology

## Abstract

*Pseudogymnoascus destructans* (*Pd*) is the causative agent of white-nose syndrome, which has resulted in the death of millions of bats in North America (NA) since 2006. Based on mortalities in eastern NA, the westward spread of infections likely poses a significant threat to western NA bats. To help prevent/reduce *Pd* infections in bats in western NA, we isolated bacteria from the wings of wild bats and screened for inhibitory activity against *Pd*. In total, we obtained 1,362 bacterial isolates from 265 wild bats of 13 species in western Canada. Among the 1,362 isolates, 96 showed inhibitory activity against *Pd* based on a coculture assay. The inhibitory activities varied widely among these isolates, ranging from slowing fungal growth to complete inhibition. Interestingly, host bats containing isolates with anti-*Pd* activities were widely distributed, with no apparent geographic or species-specific pattern. However, characteristics of roosting sites and host demography showed significant associations with the isolation of anti-*Pd* bacteria. Specifically, anthropogenic roosts and swabs from young males had higher frequencies of anti-*Pd* bacteria than those from natural roosts and those from other sex and age-groups, respectively. These anti-*Pd* bacteria could be potentially used to help mitigate the impact of WNS. Field trials using these as well as additional microbes from future screenings are needed in order to determine their effectiveness for the prevention and treatment against WNS.

## Introduction

*Pseudogymnoascus destructans* (*Pd*), the causative agent of white-nose syndrome (WNS), has been responsible for the death of more than 6 million bats in North America (NA) since 2006^1–3^. WNS is characterized by cutaneous white fungal growth on the ears, tail, wings, and muzzle of bats^4^. On the bat host, *Pd* degrades collagen, allowing the penetration of skin tissues by mycelia, resulting in lesions that form across the epidermis and invade connective tissues^5^. This leads to the perturbation of various physiological processes (electrolyte balance and hydration^6^, respiratory acidosis^7,8^, and immune system maintenance^9^), which disrupt normal hibernation behaviour, increase metabolic demands and deplete fat reserves that are vital for surviving the winter. This collection of disorders ultimately results in premature arousal from the torpid state and emaciation or death^6,10,11^. The first bat mortality from WNS was reported in 2006 near Albany, New York (NY)^12^. Since then, *Pd* has spread from this epicenter in all directions, reaching 38 US states and eight Canadian provinces^13^. The high mortality rates as a result of WNS^1,14^ have resulted in population declines in bat species across eastern North America^15,16^. The study of Vanderwolf and McAlpine^17^ in New Brunswick, Canada, reported the movement of WNS toward the northern region, with *Pd*-associated with bat mortality rates of 99% over the winter between 2011 and 2015^17^. The increasing loss of these bats has implications for the ecosystem and economy. Bats provide an ecological service as natural pest-control agents, protecting agriculture and forests by controlling insect pests^18,19^, which is also beneficial in preventing the spread of vector-borne diseases in humans^20^. Effective mitigation strategies are needed to reduce the impact of WNS on bat populations, especially in western North America, where this disease is not yet widespread.

Recent studies have presented possible avenues for the development and testing of both chemical and biological control agents against WNS^21,22^. While the use of chemical agents, including volatile organic compounds, with fungicidal/inhibitory activity can limit the growth of *Pd* mycelia and spores^23–29^, probiotics may provide a less disruptive alternative to chemical treatments. For example, applications of chemical agents in agricultural fields have shown to cause unintended adverse effects. Thus, there is increasing interest in the application of biocontrol agents, including for controlling WNS using bacteria sourced from local bat species^30^. Indeed, manipulation of the host microbiome has been successfully used to mitigate the impacts of diseases^31–33^, including for combating the fungal disease chytridiomycosis in amphibians^34,35^. Bacteria isolated from soil with anti-*Pd* activity have recently been described^36–38^ and may present promising candidates for prophylactic treatments. However, these soil-inhabiting bacteria may not be normal components of the bat microbiome, and thus, while they have anti-*Pd* activity, they may exert negative impacts on bats.

Located at the interface between the host and the environment, the surface microbiome has been shown to be capable of helping plant and animal hosts combat invasive pathogens^33,39–44^. Thus, in bats, the skin microbiome can be particularly useful for sourcing beneficial bacteria, as microbial symbionts can be passively acquired or dispersed^45^. The microbial community present on bat skin is known to be shaped by a collection of host-environment interactions, which may lead to the influx and enrichment of certain microbial taxa from external reservoirs^46–48^. Environmental reservoirs can shape the surface microbiome, especially for host bats with frequent direct contacts with roosting substrates^49^, as is the case for microbiomes of bats that show high fidelity to roosts^60^ and during winter with prolonged contacts with roosting substrates for hibernation^50^. Interestingly, the microbiomes of social organisms tend to be similar^51–54^, with the microbiomes of individuals living in a colony being more similar^55^. As a result of roosting in tight social groups, the microbiomes of bats can become synchronized^44,54^ and significantly different from bats that do not aggregate in large groups^50^. Many western bats aggregate in social groups to mate before hibernation but may disperse to winter roosts to hibernate in smaller groups or individually^56^. The skin microbial communities of the members of a social group may therefore be collectively impacted by environmental conditions instead of individual differences. This provides context upon which to evaluate factors that may contribute to the presence of key taxa capable of inhibiting *Pd* within bat skin microbiomes. At present, the potential influence of bat hosts and geographic locations on the distributions of anti-*Pd* bacteria is unknown.

While there have been reports of *Pd* and WNS in western North America, the current extent of spread is still relatively limited^12^. Identifying anti-*Pd* microbes on wings of various species of western bats may also help elucidate species-specific or geographic differences in vulnerabilities of western bats to WNS based on their wing microflora. To advance potential mitigation options for WNS in western North America, where there is high bat species diversity, we sampled bats from the western Canadian provinces of British Columbia, Alberta, and Saskatchewan to assess the anti-*Pd* activity of microbes associated with bat wings. Our main goal was to screen microorganisms from healthy wings for their anti-*Pd* activities, with an end goal of potentially augmenting their distribution on bats in nature to bolster their ability to combat *Pd* infections. As host species roost in direct contact with a variety of different environmental reservoirs (e.g., cave soil, bat box plywood substrates, rock, or tree crevices), we hypothesized bats roosting on some substrates would be more likely to harbour *Pd*-inhibiting isolates within the skin microbial community than others. Specifically, we expected bats roosting on natural substrates (i.e., rock or tree crevices) to harbour a more diverse skin microbiome than bats roosting on anthropogenic substrates (i.e., wood, concrete or steel), because the latter may contain less available nutrients for fungal growth or may be more likely to be influenced by chemicals such as plywood glues or wood treatments, or be regularly power-washed (e.g., underneath bridges^57^). We analysed the anti-*Pd* activities of bat wing bacteria in the context of geographic location, host species, roost type, sex, age, and season to identify whether any of these variables contributed significantly to the distributions of bacteria with anti-*Pd* activities.

## Results

### Culture-based identification of strains with Anti-*Pd* activity

**Figure 1:**
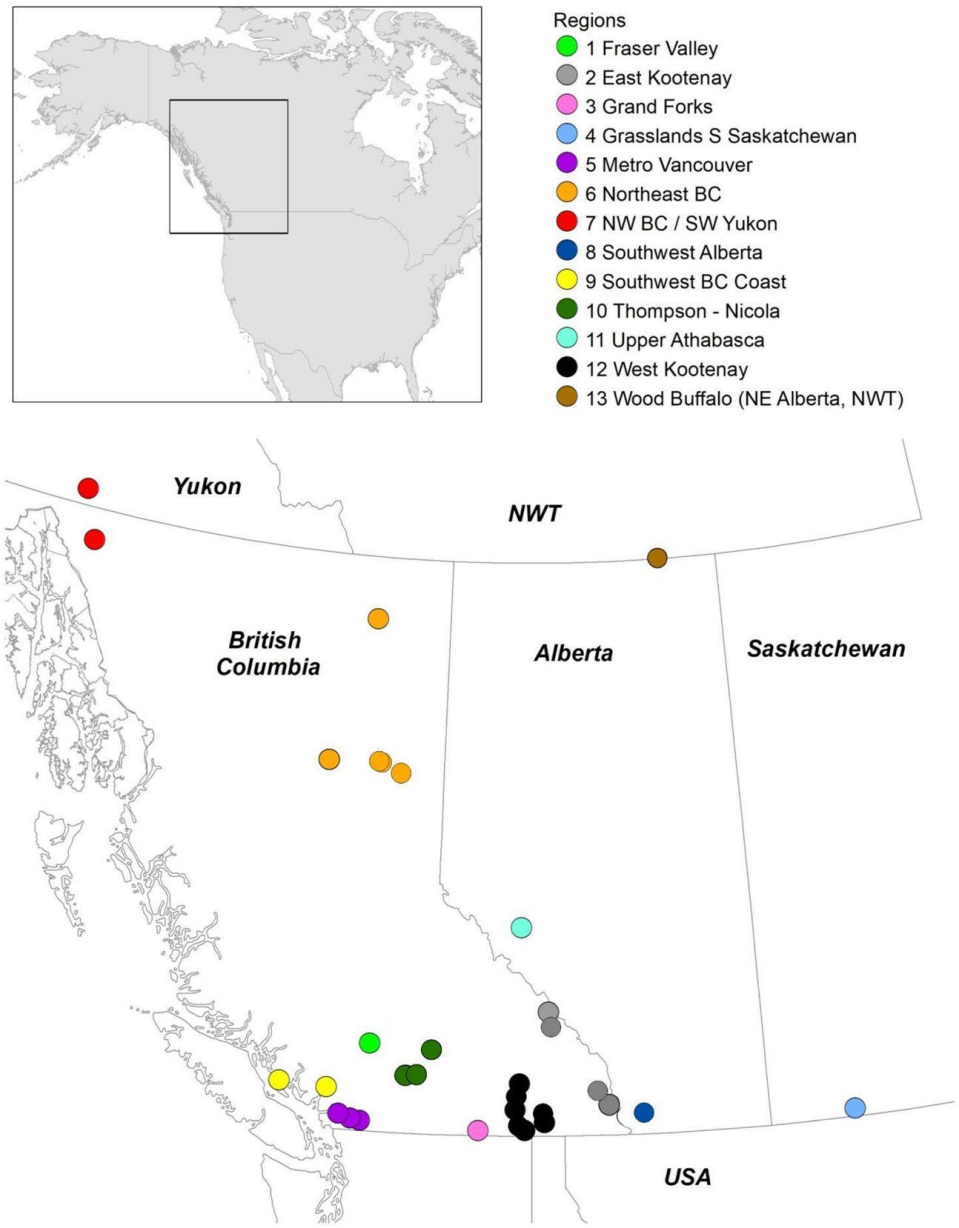
Map of the approximate sampling locations of bats surveyed in this study. Locations spanned three provinces and one territory in Western Canada. Broad geographic regions (13), based on landscape/land-use, are represented with colours (NWT = Northwest Territories). This map was made with Esri ArcMAP Geographic Information System (GIS) software version 10.8.1 and assembled using base map World Countries (Esri, Delorme Publishing Company, Inc).

**Figure 2:**
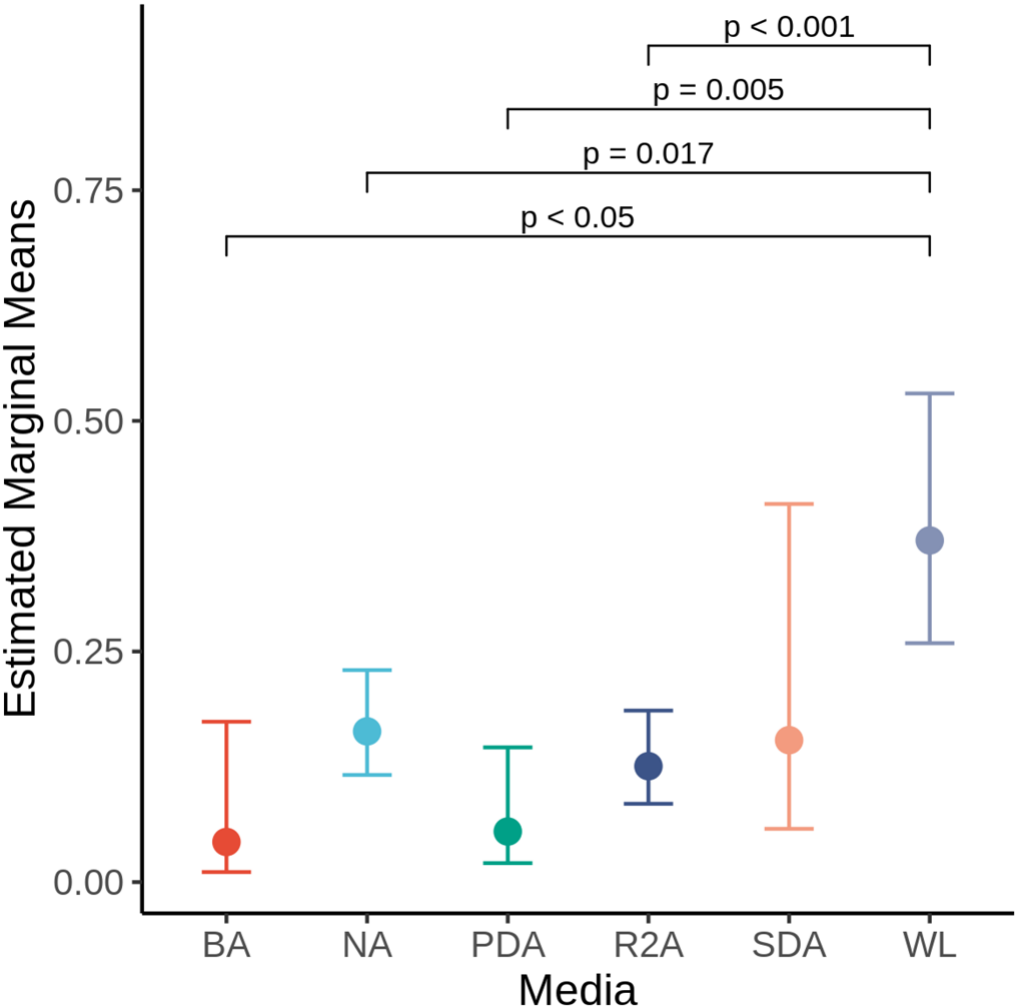
A generalized linear model was used to compare the isolation frequency of *Pd*-inhibitory bacteria with different types of microbiological media. The estimated marginal means are compared with a pairwise post hoc test, and only the resulting significant differences between groups are shown here with adjusted p values using Sidak correction.

We sampled a total of 265 wild bats, representing 13 species, using wing skin swabs from various locations across Western Canada (Fig. 1). From these skin swabs, we obtained a total of 1362 bacterial isolates, which were further assayed for anti-*Pd* activities using a culture-based screening approach^36^. More than two-thirds of the swabs collected were from 4 bat species: *Myotis lucifugus* (MYLU, n = 64), *M. yumanensis* (MYYU, n = 45), *Corynorhinus townsendii* (COTO, n = 39), and *Eptesicus fuscus* (EPFU, n = 32) bats, species that typically roost in anthropogenic structures. The remaining 79 swabs were collected from the following eight species: *M. septentrionalis* (MYSE, n = 12), *M. volans* (MYVO, n = 14), *Lasionycteris noctivagans* (LANO, n = 14), *M. californicus* (MYCA, n = 19), *Euderma maculatum* (EUMA, n = 6), *M. evotis* (MYEV, n = 18), *M. thysanodes* (MYTH, n = 1), and *M. ciliolabrum* (MYCI, n = 1), most of which rarely roost in buildings (Table 1). To help isolate microbes with different growth requirements, we used the following six types of nutrient media in our isolation: blood agar (BA), Reasoner’s 2A (R2A), potato dextrose agar (PDA), Sabouraud dextrose agar (SDA), nutrient agar (NA), and Wallerstein laboratory (WL) agar. These media encompass a range of pH values, carbon sources, and other nutrients (Table S1). Overall, the majority of bacterial isolates were isolated using NA and R2A, while a greater number of strains with *Pd* inhibition activity were isolated using WL agar (30%) compared to almost all other types of culturing media, BA, NA, PDA, and R2A (Fig. 2; p < 0.05). As not all types of microbiological media were used at the time of isolation from skin swabs, we included media as a random effect in all generalized mixed models to account for these differences in sampling. We then used these models to estimate the effects of various factors, including geographic location, host species, roost type, sex, age, and season, on the isolation of anti-*Pd* bacteria from skin swabs.

**Table 1:** A summary of the total number of bacterial strains with inhibitory activity against *Pd*.

Host species	Codes of sampled regions	Total number of swabs	Total number of Isolates	Number of isolates with partial inhibition	Number of isolates with full inhibition	Proportion of *Pd*-Inhibitory Isolates
COTO	3, 5, 12	39	226	13	3	7.08
EPFU	1, 2, 4, 6, 8, 12	32	194	14	1	7.73
EUMA	1	6	63	3	0	4.76
LANO	12	14	54	4	0	7.40
MYCA	12	19	48	2	0	4.17
MYCI	1	1	13	1	0	7.69
MYEV	2	18	57	4	0	7.02
MYLU	1, 2, 6, 7, 9, 11, 12, 13	64	291	16	3	6.53
MYSE	6, 13	12	90	4	0	4.44
MYTH	1	1	9	1	0	11.1
MYVO	5, 6, 8, 12	14	63	9	1	15.87
MYYU	2, 5, 7, 9, 10, 12	45	254	17	0	6.69

Of the 1,362 bacterial isolates screened from bat wing swabs, 7.0% demonstrated some levels of anti-*Pd* activity (Table 1). We observed that swabs from some bat species had a relatively larger proportion (MYVO, 15.87%) of the bacterial isolates with measurable anti-*Pd* activity than others (e.g., MYCA, 4.17%; and EUMA, 4.76%) (Table 1). Interestingly, swabs from two bat species where only one individual was sampled, MYTH and MYCI, high proportions of *Pd*-inhibitory isolates were found. In all following tests of geographical differences in isolation rates, we only included species that were sampled from two or more locations.

These swabs were collected from various host species and sampling locations across Western Canada. Sampling regions refer to numbers in Fig. 1 Legend.

*MYLU* *Myotis lucifugus*, *MYSE* *Myotis septentrionalis*, *MYVO* *Myotis volans*, *MYCI* *Myotis ciliolabrum*, *MYEV* *Myotis evotis*, *MYTH* *Myotis thysanodes*, *MYCA* *Myotis californicus*, *MYYU* *Myotis yumanensis*, *LANO* *Lasionycteris noctivagans*, *EPFU* *Eptesicus fuscus*, *EUMA* *Euderma maculatum*, *COTO* *Corynorhinus townsendii.*

A single generalized linear model (GLM) was constructed containing multiple factors that may influence the isolation rates of *Pd*-inhibiting isolates from skin swabs. By performing iterative addition/removal of single terms, we can estimate which factors have the largest influence on the performance of the model. The characteristics of the roost habitat and host demographics (sex/age of host species) are better predictors of isolation of anti-*Pd* bacteria than many other factors considered in this model (Table 2). The location of sampling, host species identity, and the type of media used during isolation seem to have less influence on isolation rates than the above two factors. Based on the results from this analysis, we further conducted tests to determine the influence of specific roost types and host demographics on the frequencies of isolates with anti-*Pd* activities.

**Table 2:** Iterative single-term deletions were performed on a single GLM to determine the relative influence of each variable on the frequency of *Pd*-inhibiting isolates.

	Df	Deviance	AIC
Roost type	6	232.11	454.39
Age	1	222.75	455.03
Sex	1	223.66	455.94
Season	2	226.17	456.45
Full model	–	222.64	456.92
Host species	9	243.63	459.91
Sampling location	19	264.66	460.94
Media	5	254.22	478.50

The performance of each model was compared using the Akaike information criterion (AIC).

We also tested the influences of different geographical scales, comparing the effects of local and regional groups on isolation rates (Table 3). Overall, swabs taken from hosts at nine of the 34 total locations sampled in this study did not return any bacteria with anti-*Pd* activity, and 13 sites had an anti-*Pd* bacteria isolation rate less than 10% (Table 3). We also observed an effect of sampling intensity, as locations that were sparsely sampled captured predictably fewer inhibitory isolates (p < 0.01). Despite a large sample size in several locations, some still recovered relatively few *Pd*-inhibiting isolates (i.e., Cadomin Cave, n = 155). To compare the proportions of inhibitory bacteria isolated across the various sampling locations in western Canada, subsequent analyses of geographic patterns excluded all regions with three or fewer swabs (Table 3). We were unable to distinguish differences in isolation rates between any of the locations sampled in this study while accounting for intersite differences in host species availability (GLMM with host random effect, Fig. S1). To further investigate the broader geographical patterns, we grouped sampling locations into different regions (Fig. 1) and tested the difference in isolation rates between regions with sufficient sample size (# locations >  = 3 and # species sampled ≥ 3). At this broader geographic scale, we were also unable to detect any effect on *Pd*-inhibitor isolation rates (p > 0.05; Fig. 4).

**Table 3:** Summary of the total bacterial isolates and *Pd*-inhibiting isolates identified using culturing methods for each geographic region and roost location.

Geographic regions (Fig. 1 map code)	Sampling location	Number of host Species	Number of isolates	Season	General roost characteristics	Proportion of inhibiting isolates (%)
Fraser Valley (1)	Lillooet	3	92	Summer	Underground	6.59
East Kootenay, BC (2)	Flathead Wetlands	1	5	Summer	Underground	0.00
	Hoodoo Camp	1	80	Summer	Underground	2.50
	Tent Mountain	2	12	Summer	Tree	0.00
	Yoho Ranch	2	26	Summer	Underground	11.54
Grand Forks, BC (3)	Phoenix Mine	1	15	Winter	Underground*	6.67
Grasslands Southern Saskatchewan (4)	Grasslands National Park	3	45	Summer	Anthropogenic-wood*, underground, anthropogenic-concrete*	6.52
MetroVancouver (5)	Deroche	1	118	Summer	Anthropogenic-wood*	5.93
	Minnekhada Regional Park	2	18	Summer	Anthropogenic-wood*	17.65
	Hayward	1	1	Summer	Anthropogenic-wood*	0.00
Northeast BC (6)	Alwin Holland	1	6	Fall	Tree*	0.00
	Dunlevy Creek	1	1	Fall	Underground*	0.00
	Fort Nelson	1	2	Winter	Anthropogenic-wood*	50.00
	Guyle Rock	1	11	Fall	Underground*	18.18
	Pc Bridge N Site	1	9	Fall	Tree*	11.11
	Pc Dam Viewpoint	2	77	Fall	Underground*, tree*	3.33
Northwest BC/Southwest Yukon (7)	Atlin	2	8	Summer	Anthropogenic-wood*, underground	12.50
	Tagish	1	13	Summer	Anthropogenic-wood*	0.00
Southwest Alberta (8)	Castle Provincial Park	3	28	Summer	Natural crevice, underground	0.00
Southwest Coast, BC (9)	Alice Lake Prov Park	1	14	Summer	Anthropogenic-wood*	21.43
	Powell River	1	1	Fall	Underground*	0.00
Thompson-Nicola (10)	Cold Water	2	16	Summer	Anthropogenic-wood*, underground	6.25
	Nooaitch	4	34	Summer	Anthropogenic-wood*, underground, tree	20.59
	Tranquille Barn	1	3	Fall	Anthropogenic-wood*	25.00
Upper Athabasca, Alberta (11)	Cadomin Cave	1	155	Fall, summer, winter	Underground*	3.75
West Kootenay, BC (12)	Creston Condo	1	129	Spring	Anthropogenic-wood*	6.92
	Invincible Mine	2	30	Fall	Underground*	21.74
	Jersey Mine	5	49	Fall	Underground*	7.02
	Kuskanook House	1	29	Fall	Anthropogenic-wood*	3.45
	Molly Hughes Mine	1	56	Fall, summer	Underground, anthropogenic-wood*	3.51
	Queen Victoria Mine	4	50	Fall, summer	Underground*, tree*	3.92
	Remac Mine	6	156	Fall, winter, summer	Underground*	11.11
	Beasley	1	1	Summer	Tree*	0.00
Wood Buffalo Region (NE AB, NWT) (13)	Wood Buffalo	2	72	Fall	Underground*	6.76

Roost characteristics were inferred for many captures based on examination of the capture environment. Those sites for which roost type could be confirmed directly (capture of bats as they emerged from the substrate and/or radiotelemetry) are indicated with an asterisk.

We compared rates of isolation of *Pd*-inhibiting isolates between different host species, only considering species where sufficient sampling occurred (EUMA, MYCI, and MYTH swabs were excluded). The results of this model suggest that the identity of the host species has no discernable impact on the isolation of these components of the skin microbiome (p > 0.05; Fig. 5). Wide intervals around the estimated marginal means result in no clear distinctions between the isolation rates of different host species.

**Figure 3:**
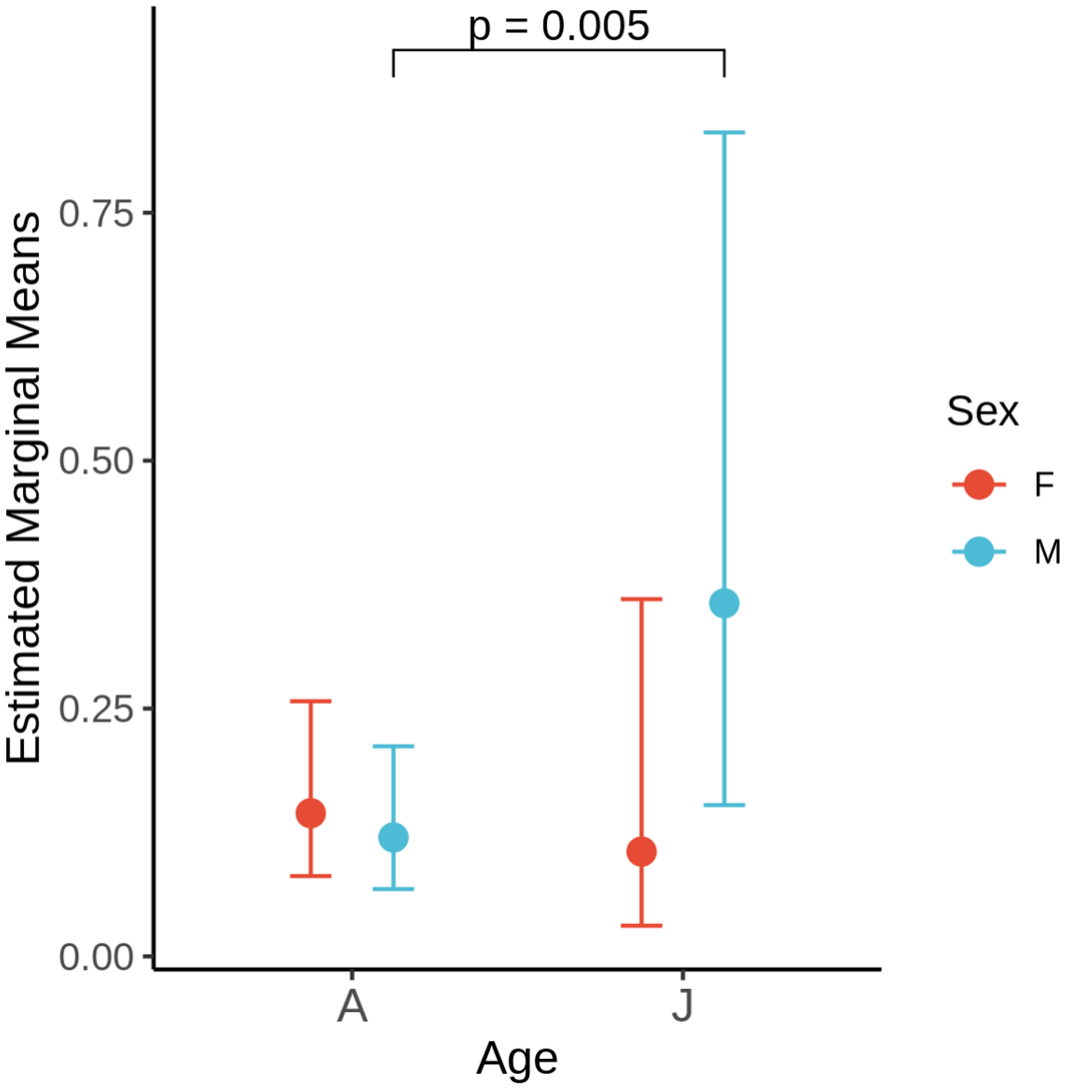
Estimated marginal means from a GLMM comparing the effects of host demographics on the isolation of bacterial strains with Pd inhibition. M = Male, A = Adult, VJ = Juvenile. P values have been adjusted (Sidak).

Similarly, we tested the potential influence of host demographics on the relative frequency of inhibitory isolates. Although juvenile bats (males, n = 9; females, n = 10) were sampled less frequently than adult bats (males, n = 115; females, n = 74), the isolation of anti-Pd isolates from adult female (n = 28), female young-of-year (n = 3) and male adult (n = 35) and male young-of-year (n = 3) bats occurred at relatively similar frequencies. In our models, we incorporated host demographic information (sex, age, and their interaction) and compared groups using estimated marginal means, accounting for differences in sample size using weights. This model demonstrates that the sampling of juvenile males has a greater number of inhibiting isolates than adult males (p <  0.01; Fig. 3), a trend that is not apparent among female hosts.

**Figure 4:**
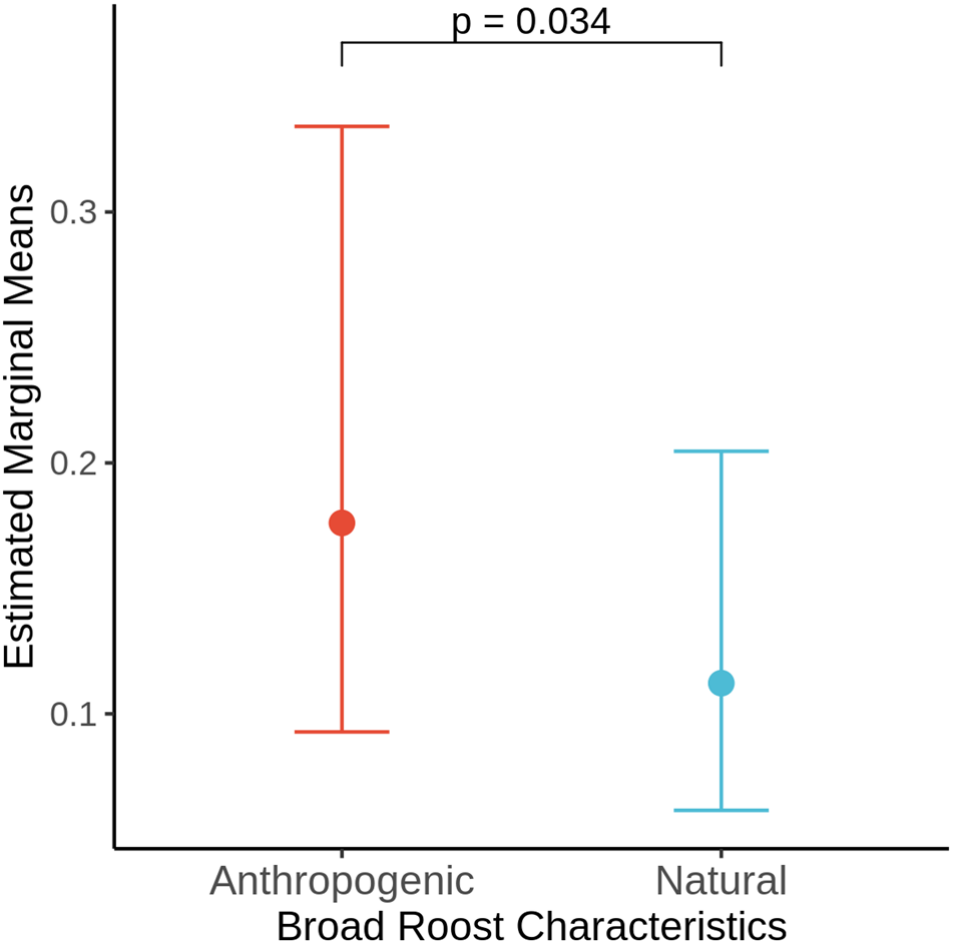
Estimated marginal means from a GLMM, testing the effects of roost characteristics, broad (**A**) and specific groups (**B**), on the prevalence of anti-Pd bacteria.

The potential influence of roosting substrate on the prevalence of *Pd*-inhibiting bacteria within the bat wing microbiome was also tested. Swabs were categorized based on the capture location and host-specific roosting behaviour (Table 4): anthropogenic (bat boxes and buildings that are heavily influenced by human activities) versus natural (rock crevice/erosion holes, tree cavity, bark crevice, cave, and mine that are less influence by human activities). To account for the availability of habitat between locations and the sampling of host species within each habitat type, we included these factors as random effects in the GLMM. Considering roosts with sufficient sample size, the results of this model show a significantly greater proportion of *Pd-*inhibiting isolates isolated from anthropogenic environments compared to those from natural niches (p < 0.05; Fig. 4).

**Table 4:** The total number of isolates collected based on roosting characteristics.

Roost characteristics	Number of isolates	Number of Pd-inhibitory isolates	Proportion of isolates with Pd-inhibitory activity (%)
**Specific**			
Concrete bridge	11	0	0.00
Building/bat box^‡^	371	34	9.16
Cave hibernaculum	227	11	4.85
Mine^‡^	354	31	8.86
Rock crevice	328	16	4.88
Tree bark/crevice	71	4	4.23
**Broad**			
*Anthropogenic	382	34	8.90
**Natural	980	62	6.33

The roosting ecology was described based on the characteristics of roosts where bats were captured at or radio-tracked to, or were derived from potential roosting habitat observed in the area (see Table 3 for further breakdown).

^‡^Although human-made, mines are rock substrates, as are caves; thus, mines were included in the natural habitat category. Bat boxes typically have plywood roosting chambers, and bats roost on various types of lumber/plywood in building roosts.

*Anthropogenic: roosts heavily influenced by human activities.

**Natural: roosts with relatively limited human influences.

**Figure 5:**
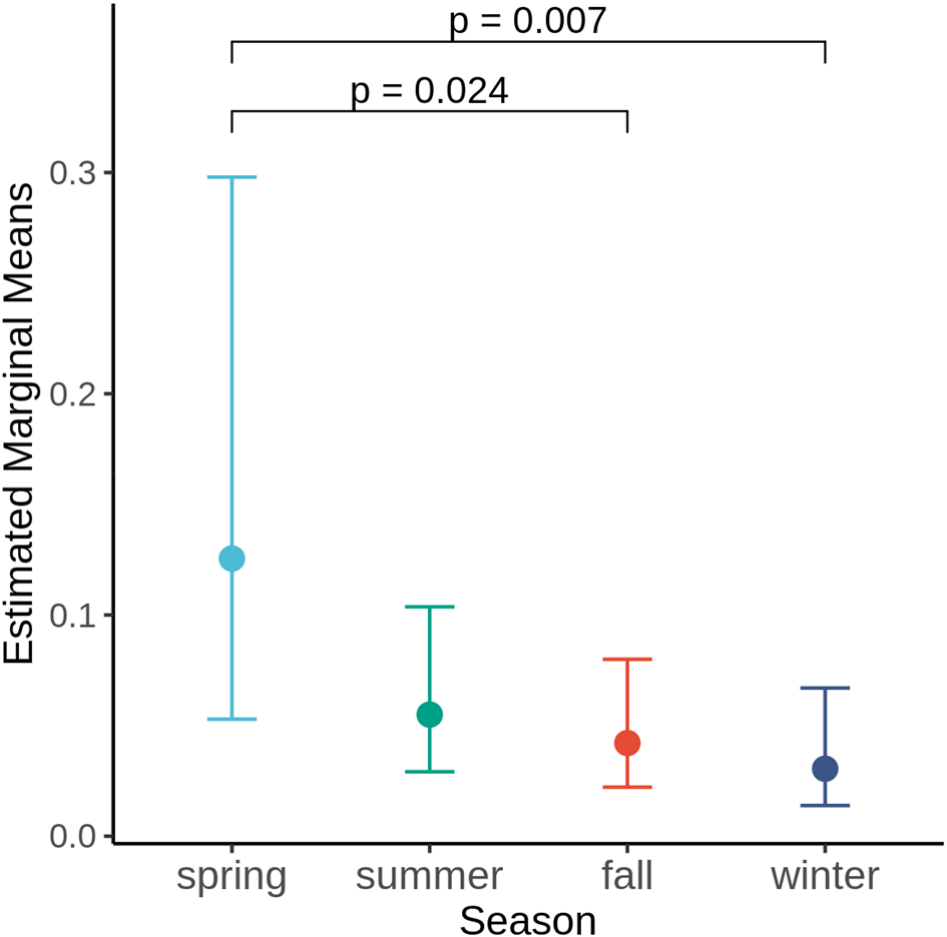
Results of a GLMM comparing the frequency of anti-*Pd* isolates collected from bats sampled during different seasons. Sampling location was included as a random factor, and the p values from the multiple comparison test of estimated marginal means for each season were adjusted based on Sidak correction.

Sampling also took place at different times of the year, with the majority taken from bats at summer roosts (n = 125), while comparably fewer taken during spring (n = 11), autumn (78), or winter (n = 51). We found more *Pd*-inhibiting isolates isolated from spring samples than from fall or winter samples (p > 0.05, Fig. 5).

### Measurements of *Pd* inhibition activity

Using a standardized challenge assay with *Pd* laboratory cultures, we measured the size of the zone of inhibition (ZOI) produced by inhibiting isolates. This allowed for a comparison of the degree of inhibition activity of bacterial isolates under laboratory conditions. Two main categories of inhibition activity were observed on SDA/PDA at optimal growth conditions for *Pd* (14 °C): complete clearing of fungal growth (full inhibition) around the bacterial colony or substantially reduced growth within the zone of inhibition around the bacterial colony (partial inhibition). Most isolates displaying anti-*Pd* activity were categorized as partial inhibiting isolates (n = 88, of 96), while eight showed full inhibitory activity.

**Figure 6:**
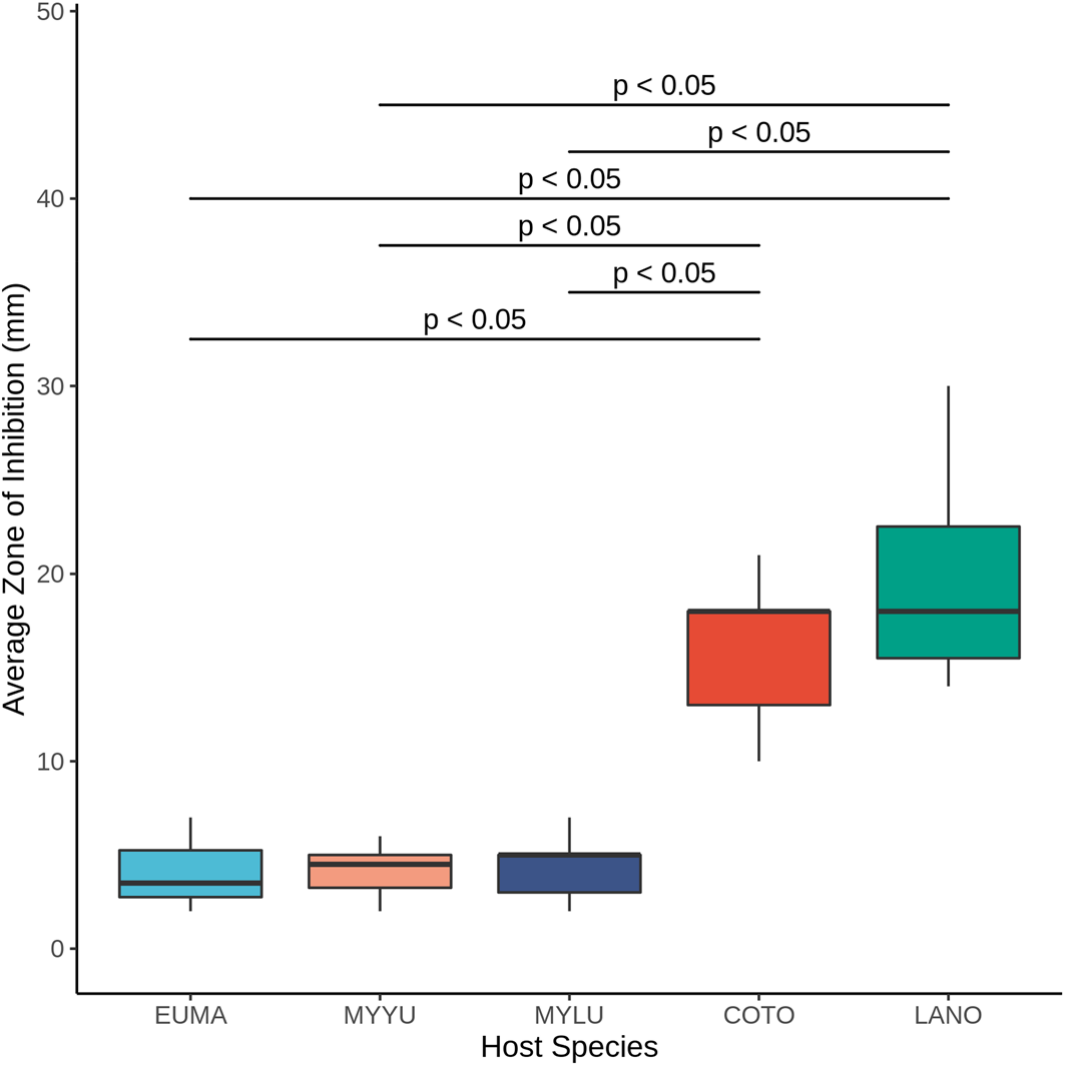
The inhibitory activity of bacterial isolates was measured in a coculture assay with *Pd*. Measurements of the average size of the zone of inhibition (ZOI) for bacterial isolates with partial inhibitory activity were compared between the different host species sampled in this study. A Wilcoxon rank test was used to compare differences in the ZOI between inhibiting isolates from different host species, only considering hosts with n ≥ 3 inhibiting isolates.

To investigate the potential contributors to the quantitative differences in inhibitory activities among anti-*Pd* isolates, we only considered bat species from which at least three inhibiting isolates were collected (Table S2). Among the host species, anti-*Pd* bacteria collected from COTO and LANO bats had significantly larger ZOIs than those isolated from EUMA, MYLU, or MYYU bats (Fig. 6; p < 0.01). We conducted similar analyses to test whether there were differences based on the geographic location of sampling (site and region) or roost substrate (natural vs anthropogenic). While the ZOIs of isolates collected from sampling locations or geographical regions did not differ significantly, we found that the isolates taken from bats that roost in mines had greater inhibitory activity (i.e., larger ZOI) than those collected from bats roosting in natural caves or artificial roost habitats (*i.e.,* buildings/bat boxes; p < 0.01; Fig. S2).

### Molecular identification of bacterial isolates with *Pd* inhibition

**Figure 7:**
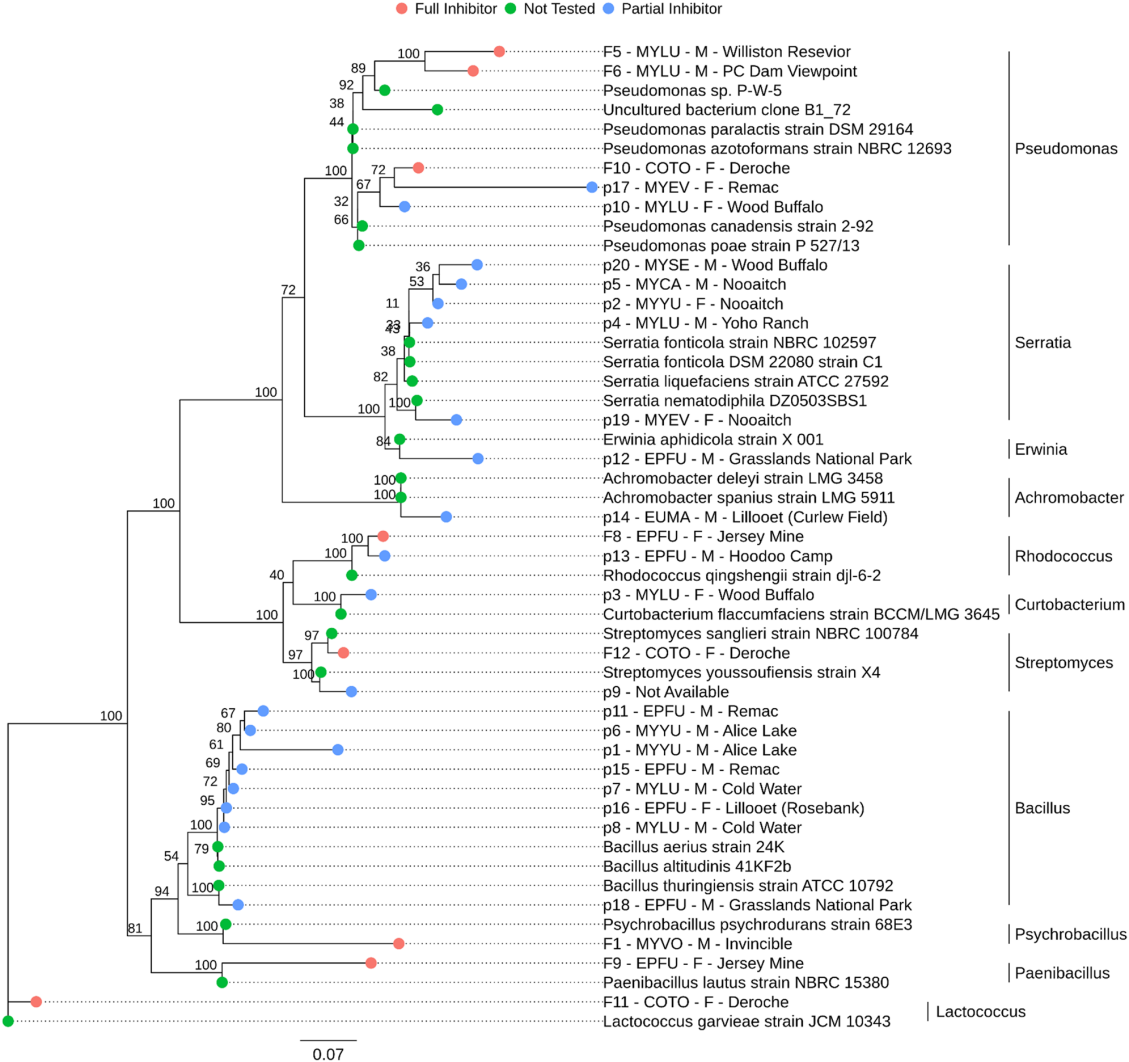
Maximum likelihood tree showing the relationships among 28 sequenced isolates with anti-Pd activities (labeled with red and blue dots) and their close known relatives from GenBank (labeled with green dots), constructed based on their 16S rRNA sequences. Tip labels include inhibitor isolate codes, host species key, host sex, and sampling location. The *Lactococcus garvieae-*type strain (JCM 10,343) is included as an outgroup. Bootstraps were generated over 10,000 replications, and scale bar units are included as the number of substitutions per site.

Based on the inhibitory activities, we selected 28 bacterial isolates representing different locations and host species for molecular identification using 16S rRNA sequencing. These included 8 full Pd-inhibiting isolates and 20 partial Pd-inhibiting isolates. Of the 28 strains, nine had less than 97% sequence similarity to 16S sequences in NCBI, suggesting that these strains may represent species that have not yet been described in such databases. However, all 28 isolates were putatively identified to generic level. In total, the 28 sequenced isolates came from 11 distinct bacterial genera: 6 Gram-positive (*Lactococcus*, *Bacillus*, *Paenibacillus*, *Curtobacterium*, *Rhodococcus*, and *Streptomyces*) and 5 Gram-negative (*Psychrobacter*, *Achromobacter*, *Erwinia*, *Serratia*, and *Pseudomonas*) (Fig. 7; Table S3). Except for the strains with putative placement within *Bacillus* (n = 8)*,* all major lineages represented in the 16S phylogenetic tree have high bootstrap support (> = 85%).

## Discussion

Using culture-based microbiological screening, we obtained a large collection of bacterial isolates with inhibitory activities against the causative agent of white-nose syndrome (WNS), *Pd*. As the skin microbiome represents an important line of protection against invasive pathogens^43,44^, the absence or decreased abundance of anti-*Pd* bacterial cells within the skin microbiome may result in increased susceptibility to *Pd*-infection^49^. These bacteria were from the skin microbiomes of bats from western Canada, and in general, there is a high diversity of bats and bat habitats in the province of B.C. Surprisingly, there was no detectable association of the isolation of strains with anti-Pd activity with geographic locations or specific host species. Although no interspecies differences in isolation rate of bacteria with anti-*Pd* activity were found, we did observe that anti-*Pd* bacteria from Silver-haired and Townsend's big-eared bats produced larger *Pd*-inhibition zones than the anti-*Pd* bacteria isolated from other bat species. While the majority of our samples were from summer months, we determined that the highest isolation of anti-*Pd* bacteria came from our spring swabs. Most of the bat species sampled in this study will roost in rock or tree crevices^56^, and many of the summer swabs of our most sampled species (Yuma Myotis, Little Brown Myotis, Townsend’s Big-eared bats) were from bats roosting in anthropogenic structures. These anthropogenic environmental samples were found to harbour a greater frequency of anti-*Pd* bacteria than natural roosting habitats. Given the rich biodiversity of bacteria in soil^58,59^, we predicted that bats roosting on wooden and concrete or steel (anthropogenic) substrates would have fewer anti-*Pd* bacteria than those roosting on natural rock or tree substrates. Additionally, samples of young males (< 1 year old) contained a higher frequency of anti-*Pd* bacteria than swabs from other groups. Below, we discuss the relevance of our results to prior findings and to the application of probiotics as potential prophylaxis in the prevention of WNS in Western bats.

### Isolation of skin bacteria using culture-assisted methods

We used several different types of culturing media to screen the microbial diversity captured in bat skin swabs. We found that a greater proportion of isolates with Pd-inhibitory activity were obtained using the WL agar (Fig. 2), a nutrient-rich medium that provides favourable growth conditions for many fungal taxa^60^. The lower pH typical of WL agar effectively inhibits the growth of many types of bacteria. The bacterial taxa capable of surviving these conditions may be more likely to encounter fungal taxa during the isolation protocol on WL agar and therefore more likely to exhibit antifungal activity. Furthermore, the production of antifungal compounds may be more common for bacteria competing with the fungi that thrive within these environments^61^. In comparison, other types of microbiological media with neutral pH could result in a lower proportion of acidophilic/acido-tolerant bacteria isolated under laboratory conditions, and therefore fewer *Pd*-inhibiting isolates were recovered from a random sample of the skin microbial community.

Culture-based screening and morphological selection of microbial isolates are not ideal for surveying the true microbial diversity present in the skin microbiome. Although in analyzing these datasets, we account for known biases in sampling design, we accept that some level of unknown bias may exist with incomplete sampling of microbial diversity and most anti-*Pd* bacteria being unidentified. However, we suggest that these sampling methods may under-represent the true diversity of the skin microbiome and of the anti-*Pd* microbial population in bats. Specifically, many bat-wing microbes are likely unable to grow on standard artificial media. In addition, our choice of culturing bacterial colonies with distinct morphologies would miss bacteria that are different but present similar colony morphologies. Furthermore, the 1,362 isolates were selected for analyses without prior knowledge of potential antifungal properties. Had they first been selected through assays exploiting their interactions with model fungi, the likelihood of obtaining anti-*Pd* bacteria would likely have increased^47,62,63^. However, despite these shortcomings, we were able to obtain a substantial number of bacterial strains with anti-*Pd* activities.

### The influence of roosting ecology and environmental factors

Across the 34 sampling locations and 13 geographic regions (Fig. 1) included in our analyses, we were unable to detect the effects of locality on the isolation of anti-*Pd* bacteria of the wing microbiome. Of all the variables we tested, the broad distinction between roosting substrates was found to explain the greatest amount of variation in the rate of isolation of *Pd*-inhibiting bacteria. There are multiple influences on the roosting ecology and habitat of bats in western Canada, where species distributions and environmental factors can be highly variable^64,65^. Specifically, bats within certain regions may have greater access to anthropogenic roosts than natural roosts. Switching from day to night roosts may also result in exposure to different substrates, with the congregation of bats at night commonly occurring at natural roosts, including rock overhangs or mines^66^. We had hypothesized that natural roosting habitats would expose bats to diverse microbial reservoirs^67^ because rock crevice, cave or tree bark roost substrates are more likely to incorporate soil or eroded sediments^67^. Close contact with roost surfaces that contain sediments rich in microbes would potentially result in a higher frequency of anti-*Pd* isolates; instead, however, our data suggested that anthropogenic roosts may expose bats to a larger number of *Pd*-inhibiting bacteria. Mines in particular were an interesting case, in that the zones of inhibition by anti-*Pd* bacteria from mines were larger than those of isolates from bats using other roost types. It is possible that frequent human entry and/or rich mineral sources in the rock influenced microbial communities at these sites. The mines from which we sampled bats in our study produced lead, zinc, silver, copper and 7 other mineral types^68^, and are infrequently visited by humans due to barriers such as gates/fences. As our sample sizes and geographic distribution of sites for many species were limited, and roost substrates for many bats could only be inferred, we believe that further investigation is needed to provide more context on the microbial reservoirs of roosting habitats with different degrees of anthropogenic influences.

We indiscriminately sampled all bats who could be captured opportunistically, with sampling occurring in all seasons depending on the site (Table 3), in order to increase the microbial diversity sampling associated with bat species in western Canada. Because our sampling occurred across different seasons and roost types and there was uneven sampling of host species both within and between sites, accurate estimates of the contributions of these factors to the frequency of anti-Pd bacteria in their wings await further investigations. Specifically, a more balanced sampling strategy across western North America is needed to investigate patterns of microbiomes associated with geography, roost types and host species. Such a study design should also consider the environmental context to test whether bats in buildings would have rich diversity of anti-*Pd* microbes across geographic areas. In particular, it would be important to take note of the types of night roosts used and roost switching among alternative day roosts in the home range of a colony of bats in buildings or bat boxes. As we have seen in our study, season could also strongly influence wing microbiomes, as bats may have shifted from different roost structures prior to being sampled or may have their wing microbiomes influenced by behaviour. For example, in our study, we found that bats captured in spring had a higher proportion of anti-*Pd* isolates. This may be due in part to the lack of grooming activity throughout winter, and thus, bats emerging in spring may have a larger number of microbes on their wings. This may also reflect the microbial diversity of the underground environments where the bats have overwintered, although where most bats in the west hibernate are not known^69^. For our study, although some bats were captured at their roost sites, many were captured during foraging or commuting, and thus, the likely roost type had to be inferred based on knowledge of each area. A thorough examination of microbial diversity comparisons may require additional tools such as radiotelemetry to identify roost switching or night-roosting behaviours and roosts.

### Host identity, behaviour, and demographics

In addition to environmental influences, host species identity and demographics were also considered possible factors influencing skin anti-*Pd* microbiome composition. The majority of skin swabs we collected were from COTO, EPFU, MYLU, and MYYU (Table 1), as these species tend to be readily accessible in anthropogenic roosts in western Canada^70,71^. While we were unable to detect species-specific effects on the frequency of anti-*Pd* isolates, there was a substantial difference in the level of *Pd*-inhibitory activity (ZOI) among isolates collected from different host species. Specifically, *Pd*-inhibiting isolates from COTO and LANO bats had greater activity (larger ZOIs) compared to those from other species. Interestingly, this coincides with what is known about the risk of WNS among eastern species, as these two species have been found to have *Pd* on their wings in eastern populations but have not developed WNS^72^.

The influence of sociality on the gut and skin microbiome in bats has previously been demonstrated^46^. Social traits are species-specific, and differences in the level of social activity may contribute to the composition of the skin microbiome^73^. For example, the species sampled in our study that use buildings in which to roost are all highly gregarious, typically roosting in large clusters, especially to raise young (MYLU, MYYU, COTO)^56^. Greater intraspecific variation in microbiome composition is expected for species that roost solitarily or in small groups compared to species that live communally. However, species roosting in smaller clusters and/or species with patchy distributions (i.e., EUMA, MYSE, MYEV, MYCI, MYCA, LANO, MYTH) were difficult to sample and thus were underrepresented in our study. For these species, increased sample sizes are needed to capture the true diversity of their wing microbiomes. Given that the host species surveyed in this study were not exclusive to a single sampling location, and because there is intraspecies variation in roost selection, additional factors varying between sampling sites may account for additional unexplained variation (e.g., environmental context including soil microbiome diversity and/or roosts selected by bats).

Across the different species and locations surveyed in this study, juvenile males showed a higher frequency of anti-*Pd* isolates than other sex- and age groups. At present, the reason for the observed differences is unknown. It’s possible that this observation was an artifact of the small sample effect from juvenile males. Given the young age, one would expect that reproductive adult females, males young-of-year and females young-of-year would have similar microbiomes within a species given that they tend to roost together during the pup-rearing season. However, studies have shown that male bats generally harbour a lower diversity of skin microbiota than females^74^, and that hormonal and developmental differences between sexes could contribute to sex-based differences in microbiome composition^75^. The lower microbiome diversity could be derived from the enrichment of specific microbes, with some of the enriched microbes containing antifungal (and thus anti-*Pd*) activities. Additional sampling and analyses are required to better understand age- and sex- based differences in microbiome structure, especially as it pertains to anti-*Pd* activity.

### Roosting environment and microbial reservoirs

We classified the types of habitat used by hosts based on characteristics of the environment, including the substrate. The rate of *Pd*-inhibitor isolation between swabs collected from bats roosting in artificial anthropogenic habitats was significantly greater than those roosting on natural substrates. Similar to natural roosting substrates, artificial substrates, such as the plywood bat boxes or the concrete or steel of buildings and bridges, likely serve as distinct microbial reservoirs, enabling the transfer of microbes between bats. Synanthropic animals are known to acquire microbes from the anthropogenic environment and, in some cases, directly from humans^76^. Although our results suggest that human-made wood/concrete/steel structures result in greater numbers of detectable/culturable inhibitory bacteria, we cannot rule out other factors differing between roosting habitats, such as the use of night roost structures, other alternate day roosts made of different substrate materials, and other influences on host behavior, such as the frequency of roost switching or the effect of clustering in large groups versus roosting in small groups or solitarily. For the purposes of this study, roost use was inferred from habitat and knowledge of species-specific ecology at the time of capture. However, direct observations in the field are necessary to conduct further tests of the impacts of the ecology of roosting sites and roosting behaviour.

### Taxonomic identification of inhibitory isolates

We selected a subset of isolates for further genetic characterization through sequencing of the 16S rRNA gene (Fig. 7). Many of the isolates that could be identified (*Lactococcus*, *Pseudomonas*, *Psychrobacillus*, *Streptomyces*, *Erwinia*, and *Achromobacte*r^77–84^) are known to grow optimally at pH 6.3–8.0. A few are acidophilic (optimal pH 4.0–5.0), including *Curtobacterium* and *Serratia*^85,86^. Finally, bacteria of the *Paenibacillus*, *Bacillus*, and *Rhodococcus* genera are commonly characterized as neutrophilic or alkaliphilic, with a broad range of optimal pH values between 6.5 and 9.5^87–89^.

Several isolates sequenced in this study likely represent novel species (Fig. 7). As cave environments can contain a wealth of currently unexplored diverse microbial populations^67^ and their components can transfer into host microbiomes, the true diversity of the skin microbiomes of cave inhabitants requires further investigation. For example, Actinobacteria, which are generally described with robust antifungal capabilities^90,91^, were infrequently isolated in our cultures (based on colony morphology data, data not shown). Our sampling methods may have selected against many Actinobacteria due to their slow growth. Indeed, Actinobacteria with anti-*Pd* activity are known to exist in caves with bats^36^. A critical comparison with culture-independent analyses is needed to determine the distribution and abundance of Actinobacteria among our bat wings^92^.

The *Pseudomonas* strains identified in this study showed the highest anti-Pd activities and are of particular interest for future probiotic applications. Members of this genus have been used to combat pathogenic fungal infections in plants, fish, or amphibian populations and have harnessed their antifungal capabilities through the addition of live cultures to the host microbiota^93–95^. Pseudomonads are frequently considered in biocontrol applications to protect against fungal infections due to their diverse metabolic capacity; many species of these bacteria are capable of producing various antifungal compounds, volatiles, antibiotics, siderophores, and mycolytic enzymes^96–100^. In addition, Pseudomonads are ubiquitous across many different environments^37^ and are commonly found within hibernacula substrate, bat skin, and bat guano^46,101–103^, which may allow bats to introduce or influence the cave roost environment in a reciprocal manner, contributing to the prevalence/stability of these components in the skin microbiome.

## Conclusion

In this study, we described a collection of 1362 microbial isolates collected from swabs of 265 wild bats in western Canada. In total, we observed inhibitory activity against *Pd* in 96 isolates. While geographic location and host species identity are not good predictors of inhibitory components of the wing microbiome, we have tentatively demonstrated that positive associations exist with certain roosting ecologies and host demographics. Additional work will be necessary to determine the underlying processes that create the bacterial community on bat wing surfaces. For example, metagenomic analyses could help determine the broader diversity of bacteria on bat wings and how they might interact with each other (based on their co-distribution patterns). Microbial interactions within the skin microbiomes of healthy bats may present a promising avenue for future development of prophylactics against WNS. We hypothesize that ecological interactions such as bat roost selection, the environmental context of roost availability together with roost switching and social behaviours may influence the culturable diversity of skin communities. Continued surveying and screening of bacterial isolates against *Pd* may help formulate effective WNS management and prevention strategies, such as the development of probiotic-based approaches for the conservation of bat populations in western Canada/the United States.

## Materials and methods

### Sampling of the bat wing microbial community

Between September 1, 2016 and August 23, 2018, we captured and swab-sampled bats at various locations in British Columbia, Alberta and Saskatchewan, Canada, including foraging areas, and roosts including mine hibernacula (in the West Kootenay region, near Salmo, Nelway and Nelson) (Table S2). We measured forearm length and used morphological features to identify the species. In the case of *M. lucifugus* versus *M. yumanensis*, echolocation calls were also recorded to differentiate species^104^. Bats were handled with disposable latex gloves and kept individually in clean cloth bags while waiting for processing. Within one hour of capture, wing microbes were collected by using rayon swabs rolled across the wing surface and placed in swab culture medium (Transwab® Amies MW170, MWE, UK). Swab samples were stored at 4 °C until they were plated on the culture media. The samples were processed for isolation as soon as possible. All bats were released on site, and all methods and protocols followed Canadian Council on Animal Care guidelines, as outlined in the wildlife capture permit issued to C. Lausen from BC Ministry of Environment (permit #MRCB15-163558), Ministry of Forests, Lands, Natural Resource Operations and Rural Development, TRU Animal Care Committee. The study is reported in accordance with ARRIVE guidelines (https://arriveguidelines.org).

### Culture-based screening of bat skin swabs

We screened the anti-*Pd* activity of all morphologically distinct microbial colonies that grew from bat wing swabs and cultured under similar laboratory conditions. Screening of microbial isolates followed a consistent protocol. Microbes collected from bat wings were transferred via swabs onto the following six media: blood agar (BA), Reasoner’s 2A (R2A), potato dextrose agar (PDA), Sabouraud dextrose agar (SDA), nutrient agar (NA), and Wallerstein laboratory (WL) agar. These inoculated plates were incubated at 30 °C for 24–48 h, at which point morphologically distinguishable colonies were subcultured, and purified colonies were further screened for anti-*Pd* activity. The 30 °C was chosen because it’s the threshold temperature for many bats being active.

The anti-*Pd* activity method used in this study was reported previously^105^. The cultivation, isolation of *Pd* and preparation of the *Pd*-seeded SDA plates were performed following protocols established previously^29,106^, using the *Pd* strain ATCC 20,631–21 as the test strain^107,108^. Spores of *Pd* were isolated from *Pd* cultivated at 15 °C for 14 days on SDA media. As previously described by Cornelison et al.^24^, spore suspensions were created by scraping large quantities of mycelial growth off solid media and filtering the suspension through sterile glass wool. The concentration of spores in solution was quantified with a hemocytometer, and spore solutions were stored at 4 °C for no longer than 4 weeks. From standardized cell suspensions of 7 × 10^6^ *Pd* spores/mL, 555 μl of spore solution was inoculated onto SDA media plates using a sterile glass rod.

The purified bacterial isolates from the bat wing swabs were picked aseptically and point inoculated on *Pd-*seeded SDA plates using the agar plug diffusion method^29,106^. Following inoculation, the plates were incubated at 15 °C and observed periodically for anti-*Pd* activities, as reflected by the zone of inhibition (ZOI). The ZOIs were measured in millimeters as the total diameter of the zone of growth inhibition. We characterized the inhibition as “partial”, in which inhibition is present but the isolate does not completely prevent *Pd* germination within the ZOI, and “full”, in which no *Pd* spores germinate within the ZOI^106^.

### Molecular identification and phylogenetics of isolates with Anti-*Pd* activity

Bacterial genomic DNA was extracted using a modified CTAB method^109^. The isolated DNA was PCR amplified for the complete 16S rRNA region (protocol of Ghosh et al.^29^ using universal primers, 8F and 1492R^110,111^), followed by Sanger sequencing at Mobix Lab (Hamilton, Ontario). The similarity of sequences to known species was compared against the NCBI 16S rRNA bacterial database^112^ using BLAST+^113^. We included the sequences of the closest related species based on BLAST search criteria. The full dataset, including these representative sequences and the 16S sequences generated from both full and partial *Pd*-inhibiting isolates, was then aligned using MAFFT^114^ prior to building a maximum likelihood tree using RAxML^115^, bootstrapping values generated over 10,000 iterations.

### Statistical analyses of isolation rates

All statistical analyses were conducted in R (V4.0.1^116^). We constructed a preliminary GLM to test the effect of all factors on the total inhibitor proportion (inhibiting isolates/total) and included an offset by sample size. Using the *dropterm* function in the *MASS* package^117^, we performed all iterations of single-term deletions on the complete GLM and assessed the performance of each model using the Akaike information criterion (AIC). To address multiple different hypotheses on the impacts of host/site characteristics on the frequencies of strains with *Pd*-inhibiting activity, we constructed a series of generalized linear mixed models (GLMMs) using the package *lme4*^118^. All GLMMs used a Poisson link function, as our observations included counts of observed *Pd*-inhibition activity within distinct isolation trials; offset of the number of isolates obtained per swab within each group was also included in all models. Within select individual models that we constructed to isolate the effects of specific variables, we included random effects to control for confounding impact of other variables. Levels of some factors were excluded in cases where certain host species or sampling locations were undersampled, resulting in infinitely large margins of error in our estimates. Estimates of marginal means, credible intervals, and Sidak adjusted p values were generated using the functions available within the R package *emmeans*^119^. Additional statistical analyses of ZOI measurements involved pairwise Kruskal-Wallace tests, visualized using *compare_means* within the R package *ggpubr*^120^.

## Supplementary Information

Supplementary Information.

## Data Availability

We confirm that all the data associated with this manuscript are freely available and are presented either within the main manuscript file or in the Supplementary Materials.
