# Supplementary material for: Microbial isolates with Anti-Pseudogymnoascus destructans activities from Western Canadian bat wings
Source: Sci Rep. 2022 Jun 14;12:9895. doi: 10.1038/s41598-022-14223-9 (PMC9198084; doi:10.1038/s41598-022-14223-9)
Supplement: Supplementary file 1 — Supplementary Information. [file 41598_2022_14223_MOESM1_ESM.docx]

# Supplementary Information

###

### Table S1. Number of anti-*Pd* isolates found on each type of medium and percentage of bacteria with inhibitory activities.

| **Media** | **Media Characteristics** [^117,118^](https://paperpile.com/c/sZ72VZ/ycMy+3Fc4) | **pH** [^117,118^](https://paperpile.com/c/sZ72VZ/ycMy+3Fc4) | **Number of isolates** | **Number of isolates with Inhibition** | **Percentage of bacteria with anti-*Pd* activity** |
| --- | --- | --- | --- | --- | --- |
| Nutrient Agar | cultivation of organisms that are not demanding in their nutritional requirements | 7.3 ± 0.2 | 526 | 33 | 6.27% |
| Wallerstein Laboratory Agar | Isolation and enumeration of yeasts, moulds and bacteria in the brewing process | 5.5 ± 0.2 | 119 | 28 | 23.53% |
| Reasoner’s 2A | A medium of low nutritional content for use with isolation of bacteria from water samples | 7.2 ± 0.2 | 492 | 25 | 5.08% |
| Potato Dextrose Agar | selective medium for fungi and yeasts. and inhibits many species of bacteria | 5.6 ± 0.2 | 106 | 4 | 3.77% |
| Sabouraud Dextrose Agar | selective medium for fungi and yeasts. and inhibits many species of bacteria | 5.6 ± 0.2 | 56 | 4 | 7.14% |
| Blood Agar | cultivate a wide range of microorganisms of clinical significance | 7.4 ± 0.2 | 63 | 2 | 3.17% |
|  | | | Total: 1362 | Total: 96 | Average: 7.05% |

###

### Table S2: Sampling location and sample size for each host species. See Figure 1 for map codes (regions).

| Site Name | Map Region | COTO | EPFU | EUMA | LANO | MYCA | MYCI | MYEV | MYLU | MYSE | MYTH | MYVO | MYYU | Site Totals |
| --- | --- | --- | --- | --- | --- | --- | --- | --- | --- | --- | --- | --- | --- | --- |
| Lillooet | 1 | - | 2 | 6 | - | - | - | - | - | - | 1 | - | - | 9 |
| Flathead FSR Wetlands | 2 | - | - | - | - | - | - | - | 2 | - | - | - | - | 2 |
| Hoodoo Camp | 2 | - | 6 | - | - | - | - | - | - | - | - | - | - | 6 |
| Tent Mountain | 2 | - | - | - | - | - | - | 2 | 2 | - | - | - | - | 4 |
| Yoho Ranch | 2 | - | - | - | - | - | - | 1 | 2 | - | - | - | - | 3 |
| Phoenix Mine | 3 | 3 | - | - | - | - | - | - | - | - | - | - | - | 3 |
| Grasslands National Park | 4 | - | 8 | - | - | - | 1 | 2 | - | - | - | - | - | 11 |
| Deroche | 5 | 17 | - | - | - | - | - | - | - | - | - | - | - | 17 |
| Hayward | 5 | - | - | - | - | - | - | - | - | - | - | - | 2 | 2 |
| Minnekhada Regional Park | 5 | 1 | - | - | - | - | - | - | - | - | - | 1 | - | 2 |
| Alwin Holland | 6 | - | - | - | - | - | - | - | - | 1 | - | - | - | 1 |
| Dunlevy Creek | 6 | - | - | - | - | - | - | - | - | - | - | 1 | - | 1 |
| Fort Nelson | 6 | - | 1 | - | - | - | - | - | - | - | - | - | - | 1 |
| Guyle Rock (Williston Reservoir) | 6 | - | - | - | - | - | - | - | - | - | - | 1 | - | 1 |
| Peace Canyon | 6 | - | - | - | - | - | - | - | - | 1 | - | - | - | 1 |
| Peace Dam Viewpoint | 6 | - | - | - | - | - | - | - | 5 | 2 | - | - | - | 7 |
| Atlin | 7 | - | - | - | - | - | - | 2 | 4 | - | - | - | - | 6 |
| Tagish | 7 | - | - | - | - | - | - | - | 4 | - | - | - | - | 4 |
| Castle Provincial Park | 8 | - | 4 | - | - | - | - | 2 | - | - | - | 4 | - | 10 |
| Alice Lake Provincial Park | 9 | - | - | - | - | - | - | - | - | - | - | - | 11 | 11 |
| Powell River | 9 | - | - | - | - | - | - | - | 1 | - | - | - | - | 1 |
| Cold Water | 10 | - | - | - | - | - | - | 1 | 1 | - | - | - | - | 2 |
| Nooaitch | 10 | - | - | - | - | 1 | - | 4 | 1 | - | - | - | 2 | 8 |
| Tranquille Barn | 10 | - | - | - | - | - | - | - | 2 | - | - | - | - | 2 |
| Cadomin Cave | 11 | - | - | - | - | - | - | - | 30 | - | - | - | - | 30 |
| Beasley | 12 | - | - | - | - | 1 | - | - | - | - | - | - | - | 1 |
| Creston Condo | 12 | - | - | - | - | - | - | - | - | - | - | - | 11 | 11 |
| Invincible Mine | 12 | - | - | - | - | - | - | - | - | - | - | 3 | 3 | 6 |
| Jersey Mine | 12 | 1 | 3 | - | - | 1 | - | - | - | - | - | 3 | - | 8 |
| Kuskanook House | 12 | - | - | - | - | - | - | - | - | - | - | - | 7 | 7 |
| Molly Hughes Mine | 12 | - | - | - | - | - | - | - | - | - | - | - | 9 | 9 |
| Queen Victoria Mine | 12 | 3 | 2 | - | 5 | 3 | - | - | - | - | - | - | - | 13 |
| ReMac Mine | 12 | 14 | 6 | - | 9 | 13 | - | 4 | - | - | - | 1 | - | 47 |
| Wood Buffalo Caves | 13 | - | - | - | - | - | - | - | 10 | 8 | - | - | - | 28 |
| Species Totals | | 39 | 42 | 6 | 14 | 19 | 1 | 18 | 64 | 12 | 1 | 14 | 45 | 265 |

*Species codes: MYLU = *Myotis lucifugus*, MYSE = *Myotis septentrionalis*, MYVO = *Myotis volans*, MYCI = *Myotis ciliolabrum*, MYEV = *Myotis evotis*, MYTH = *Myotis thysanodes*, MYCA = *Myotis californicus*, MYYU = *Myotis yumanensis*, LANO = *Lasionycteris noctivagans*, EPFU = *Eptesicus fuscus*, EUMA = *Euderma maculatum*, COTO = *Corynorhinus townsendii*

### Table S3: Results of sequencing of 16S V3-V4 regions for select inhibitory isolates. The approximate taxonomic identity was based on comparison of these sequences to the curated 16S NCBI database using BLAST.

| **Isolate**  **ID** | **Identity of Closest Species** | | **Percent**  **Identity** | **Alignment**  **Length** | **Mismatch** | **Approximate E-score** | **Bits** |
| --- | --- | --- | --- | --- | --- | --- | --- |
|  | **GenBank Accession** | **Name** |  |  |  |  |  |
| F4 | NR_152014.1 | *Achromobacter deleyi* | 93.29 | 328 | 20 | 0 | 486 |
| F12 | NR_041417.1 | *Streptomyces sanglieri* | 98.31 | 1421 | 19 | 0 | 2486 |
| F3 | NR_025686.1 | *Achromobacter spanius* | 98.01 | 1409 | 22 | 0 | 2444 |
| F11 | NR_113268.1 | *Lactococcus garvieae* | 97.51 | 1443 | 25 | 0 | 2455 |
| F2 | NR_025686.1 | *Achromobacter spanius* | 92.25 | 1394 | 97 | 0 | 1967 |
| F10 | NR_113600.1 | *Pseudomonas azotoformans* | 96.37 | 1405 | 39 | 0 | 2303 |
| F1 | NR_025409.1 | *Psychrobacillus psychrodurans* | 86.25 | 1411 | 157 | 0 | 1496 |
| F9 | NR_112724.1 | *Paenibacillus lautus* | 88.24 | 1437 | 132 | 0 | 1687 |
| F8 | NR_115708.1 | *Rhodococcus qingshengii* | 97.72 | 1405 | 19 | 0 | 2405 |
| F7 | NR_156987.1 | *Pseudomonas paralactis* | 98.34 | 661 | 7 | 0 | 1157 |
| F5 | KC991307.1 | *Pseudomonas sp. P-W-5* | 92.09 | 909 | 1 | 0 | 637 |
| F6 | EU789853.1 | Uncultured bacterium clone B1_72 | 92.28 | 1102 | 1 | 0 | 1318 |
| p8 | NR_042337.1 | *Bacillus altitudinis* 41KF2b | 99.45 | 1446 | 2 | 0 | 2621 |
| p16 | NR_042337.1 | *Bacillus altitudinis* 41KF2b | 99.38 | 1439 | 4 | 0 | 2603 |
| p7 | NR_042337.1 | *Bacillus altitudinis* 41KF2b | 98.82 | 1442 | 8 | 0 | 2560 |
| p15 | NR_042337.1 | Bacillus altitudinis 41KF2b | 97.92 | 1443 | 19 | 0 | 2488 |
| p6 | NR_118439.1 | *Bacillus aerius* | 98.38 | 1421 | 9 | 0 | 2484 |
| p14 | NR_025686.1 | *Achromobacter spanius* | 93.99 | 1415 | 26 | 0 | 2089 |
| p5 | NR_116808.1 | *Serratia fonticola* DSM 22080 | 97.08 | 1402 | 30 | 0 | 2351 |
| p13 | NR_115708.1 | *Rhodococcus qingshengii* | 98.2 | 1385 | 21 | 0 | 2416 |
| p4 | NR_114156.1 | *Serratia fonticola* | 97.37 | 647 | 11 | 0 | 1099 |
| p12 | NR_104724.1 | *Erwinia aphidicola* | 93.52 | 1420 | 81 | 0 | 2102 |
| p20 | NR_121703.1 | *Serratia liquefaciens* | 95.76 | 1416 | 47 | 0 | 2281 |
| p3 | NR_025467.1 | *Curtobacterium flaccumfaciens* | 97.23 | 1410 | 27 | 0 | 2377 |
| p11 | NR_118439.1 | *Bacillus aerius* | 97.55 | 1429 | 23 | 0 | 2435 |
| p19 | NR_044385.1 | *Serratia nematodiphila* DZ0503SBS1 | 97.04 | 1419 | 29 | 0 | 2375 |
| p2 | NR_116808.1 | *Serratia fonticola* DSM 22080 | 97.62 | 1426 | 20 | 0 | 2433 |
| p10 | NR_028986.1 | *Pseudomonas poae* | 96.74 | 1411 | 35 | 0 | 2340 |
| p18 | NR_114581.1 | *Bacillus thuringiensis* | 98.40 | 1439 | 11 | 0 | 2519 |
| p1 | NR_118439.1 | *Bacillus aerius* | 90.06 | 1429 | 117 | 0 | 1831 |
| p9 | NR_116980.1 | *Streptomyces youssoufiensis* | 97.64 | 1397 | 23 | 0 | 2388 |
| p17 | NR_156852.1 | *Pseudomonas canadensis* | 97.48 | 713 | 10 | 0 | 1210 |

###


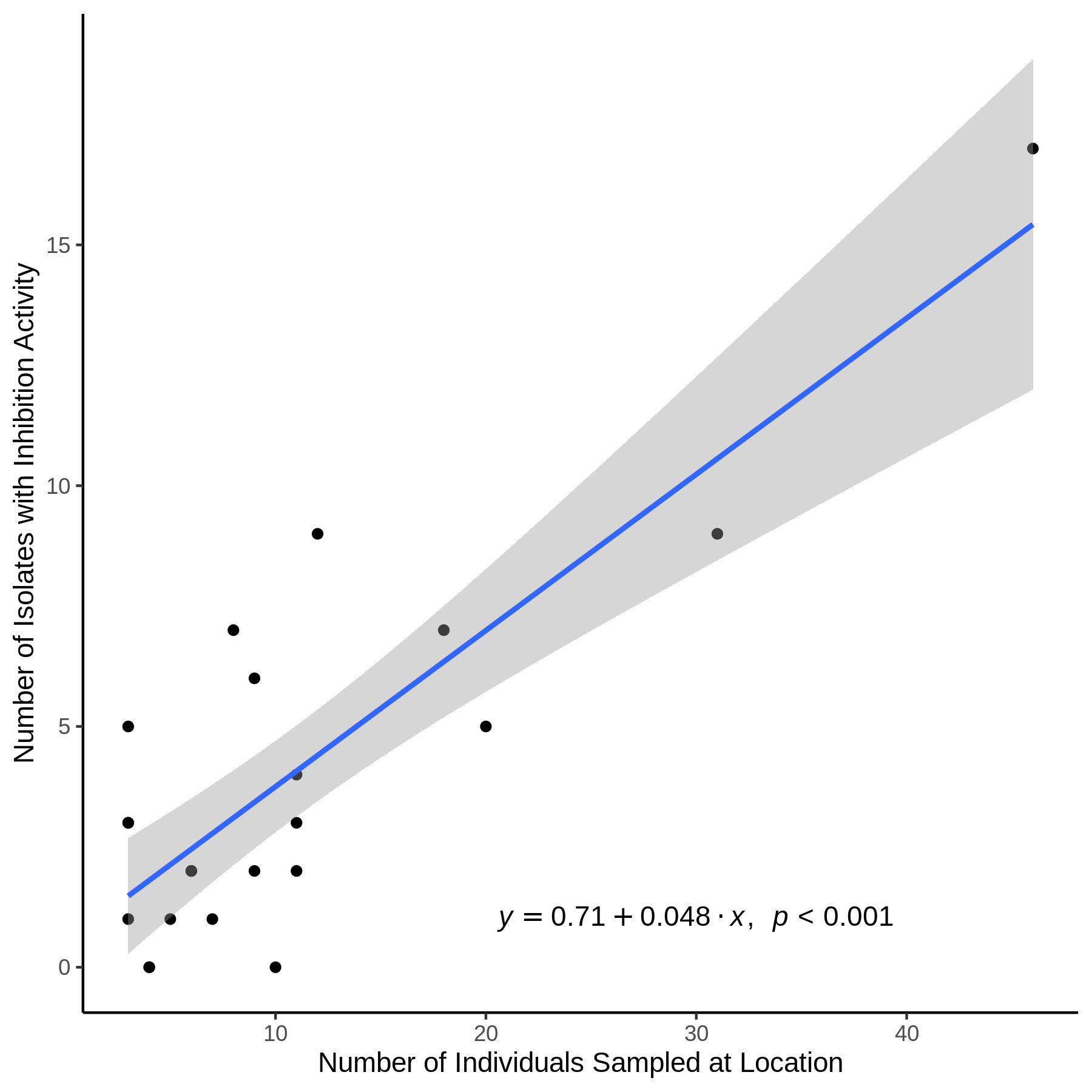


### Figure S1: The results of a GLM showing the relationship between the number of bats sampled at a site and the number of isolates recovered with *Pd* inhibition.


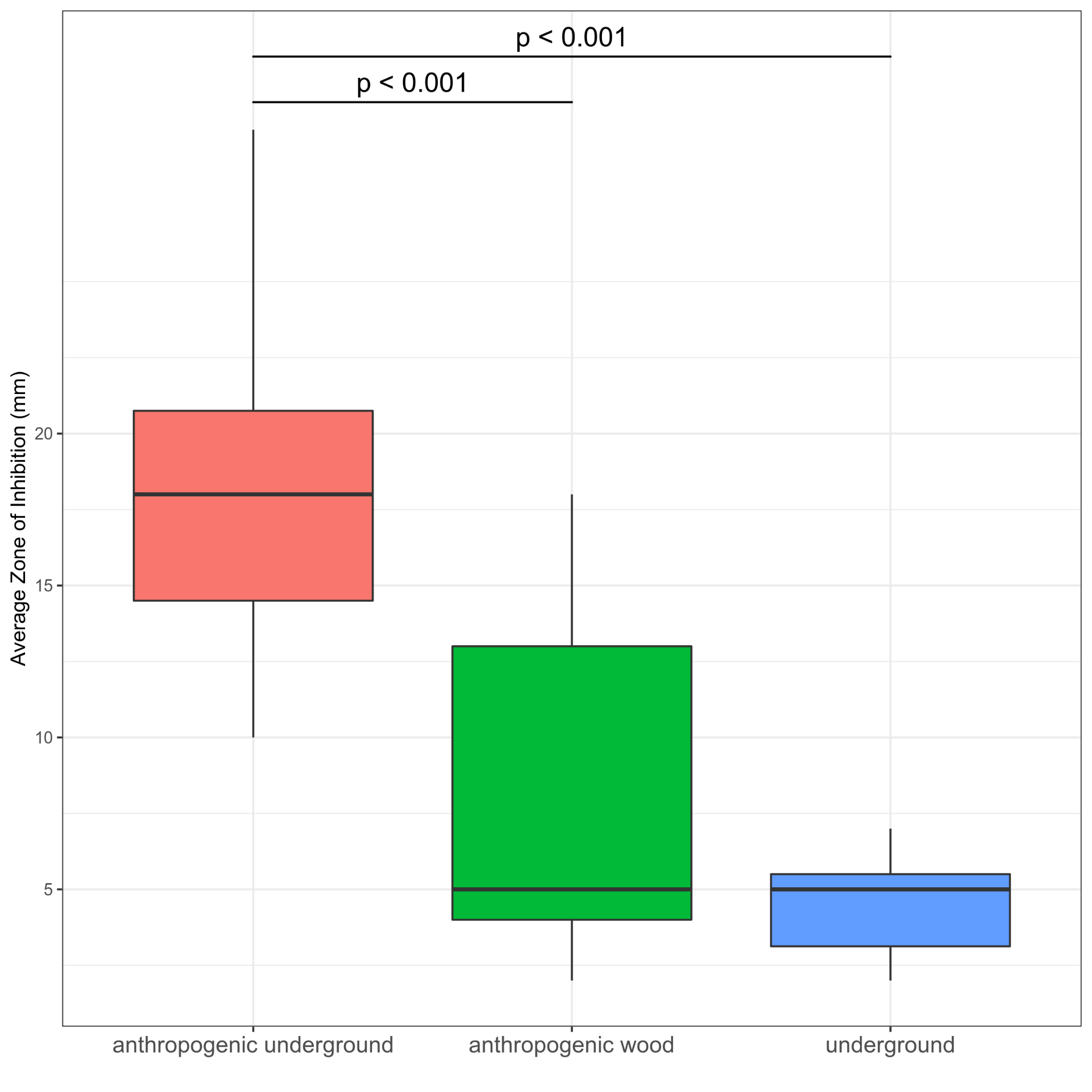


### Figure S2: The size of zones of inhibition for *Pd* inhibitors sourced from hosts inhabiting different roost types.
